# Exploring the Photocatalytic Efficiency of Gold Nanoparticles Deposited on Ni-Al-Zr-Layered Double Hydroxides for Selective Glucose Oxidation

**DOI:** 10.3390/molecules30010013

**Published:** 2024-12-24

**Authors:** Nihel Dib, Frédéric Sauvage, Lucie Quéhon, Khadidja Khaldi, Sumeya Bedrane, José Juan Calvino, Redouane Bachir, Ginesa Blanco, Gwladys Pourceau

**Affiliations:** 1 Laboratory of Catalysis and Synthesis in Organic Chemistry (LCSCO), University of Tlemcen, BP 119, Tlemcen 13000, Algeria; 2 Departamento de Ciencia de los Materiales, Ingeniería Metalúrgica y Química Inorgánica, Facultad de Ciencias, Universidad de Cádiz, Campus Río San Pedro, E-11510 Puerto Real, Cádiz, Spain; 3 Instituto Universitario de Investigación en Microscopía Electrónica y Materiales (IMEYMAT), Universidad de Cádiz, Campus Río San Pedro, E-11510 Puerto Real, Cádiz, Spain; 4 Laboratoire de Réactivité et Chimie des Solides (LRCS), UMR CNRS 7314, Université de Picardie Jules Verne, Hub de L’énergie, 15 rue Baudelocque, FR-80000 Amiens, France; 5 Centre de Recherche Scientifique et Technique en Analyses Physico-Chimiques CRAPC, BP 384, Tipaza 42004, Algeria; 6 Laboratoire de Glycochimie et des Agroressources d’Amiens (LG2A) UR 7378, Université de Picardie Jules Verne, FR-80000 Amiens, France

**Keywords:** photocatalysis, layered double hydroxides, gold nanoparticles, glucose oxidation

## Abstract

Confronting escalating challenges in energy security and environmental sustainability has intensified interest in renewable sources for fuels and chemicals. Among the most promising alternatives, sugars derived from biomass are emerging as a cornerstone in advancing an environmentally sustainable economy. Within this framework, the development of sunlight-driven carbohydrate oxidation is of significant interest, as it enables the production of a broad spectrum of high-value, bio-sourced chemicals through eco-friendly processes. Gold nanoparticles (Au NPs) immobilized on inorganic supports have demonstrated considerable potential in this area, although the methodology still requires further exploration. In this study, we explored the selective oxidation of glucose into the corresponding gluconic acid salt in presence of a novel Au/Ni-Al-Zr-layered double hydroxide (LDH) photocatalyst under standardized A.M. 1.5 G light illumination. To optimize the photocatalytic conditions, an experimental plan is herein proposed, highlighting the critical influences of both catalyst loading and pH. In optimal conditions, the Au catalyst demonstrated a high efficiency, achieving 87% glucose conversion and 100% selectivity towards gluconic acid in only 90 min. By means of long-pass filters to select the incident light energy to the photocatalytic reactor, we evidenced that the charge transfer processes were occurring from the Ni-Al-Zr LDH support to the gold nanoparticles, thus opening new directions towards further photocatalyst modifications. This work underlines the potential of Au/LDH materials for sunlight-driven photocatalysis and provides a pathway for the sustainable production of high-value chemicals from renewable biomass sources.

## 1. Introduction

The increasing issues related to the depletion of fossil resources and the pollution generated by their use have sparked great enthusiasm for the production of renewable chemicals, especially those coming from carbohydrates, which account for 75% of biomass [1]. Among the different organic transformations carbohydrates can undergo, C1-oxidation is of particular interest since it leads to oxidized derivatives, such as aldonic acids, salts, esters, or lactones, which are widely used in the chemical, pharmaceutical, food, and cosmetic industries [2]. However, the traditional methods used for the synthesis of oxidized derivatives typically exhibit several inherent limitations [3,4]. These include potential environmental and human toxicity due to the use of harsh reagents; limited reaction selectivity, leading to the formation of undesired by-products; and challenges associated with the efficient separation of the desired products from the reaction mixture. However, the synthesis of bio-based molecules derived from sugars as potential alternatives to petrochemical compounds is meaningful only if it is carried out using sustainable processes.

In this context, photocatalytic transformation is regarded as one of the most promising sustainable and environmentally friendly procedures, especially when realized under realistic conditions [5]. As a clean, a renewable, and an abundant source of energy, the direct utilization of sunlight is of great interest in limiting the constant rise in fossil fuels demand. The concept of harnessing solar energy for organic transformations, inspired by natural photosynthesis, dates back over a century to Ciamician’s visionary work, “*The Photochemistry of the Future*”, which proposed replacing fossil fuels with sunlight for chemical production [6]. The paramount challenge for chemists is to develop an efficient method for the artificial conversion of solar energy using either biochemical, organic, or inorganic materials. In the latter case, although considerable efforts have been devoted to the design of semiconducting (SC) materials capable of achieving high photocatalytic efficiency and selective charge transfer processes under visible light exposure, rather than relying on the lower photon flux of UV light, the most promising photocatalyst materials developed to date suffer from low oxidative selectivity and limited spectral absorption in the UV region due to their excessively large bandgap. This is the case in the benchmark anatase TiO_2_ semiconductor, as its bandgap of 3.20 eV restricts light absorption and conversion to wavelengths of below 410 nm, thus accounting for, at best, only 5% of total solar irradiance. This is notably the first and most widely used material for the photocatalytic transformation of glucose [7,8,9,10,11,12]. In these studies, the authors reported that the photocatalytic oxidation of glucose led to mixtures of aldoses such as arabinose, erythrose, and glyceraldehyde and oxidized sugars such as arabinonic, gluconic, and glucaric acids. Besides lacking selectivity, these reactions are often carried out under UV light or illumination conditions that are unrealistic with respect to real outdoor applications (high light power). However, the ultimate goal for photocatalysis is the development of a catalytic system that can ultimately work within a solar biorefinery concept using sunlight as its energy source. To this end, it is paramount to develop photocatalytic systems that are able to drive photo-chemical reactions by using visible light. For this, it is fundamental that we develop photocatalytic systems that are active under visible light, with materials such as modified C_3_N_4_ [13], Zn_1−x_Cd_x_S [14], or metal-doped TiO_2_ [15], which have been successfully developed for this purpose.

In particular, the use of gold nanoparticles (Au NP) as oxidation catalysts has been well-known for over 20 years, since the first paper by Biella [16], to enhance the eco-efficiency of carbohydrate transformation while simultaneously increasing selectivity for the desired product and reducing both time and waste [17,18,19]. Moreover, these oxidizing properties have also been leveraged for the detection of reductive organic molecules such as tramadol and hydrazine [20,21] or Cd^2+^ cations [22]. Thanks to their localized surface plasmon resonance (LSPR), and if deposited on a suitable semi-conducting material, Au NPs can also exhibit interesting photocatalytic properties, even under realistic incident light illumination conditions [23,24,25,26,27,28]. Nevertheless, in all our investigations concerning the gold-catalyzed photo-oxidation of sugars, as well as in a majority of the studies reported in the literature, maintaining alkaline conditions has proven essential to avert catalyst deactivation.

Otherwise, two-dimensional inorganic layered double hydroxides (LDHs), also known as anionic clays, have attracted immense interest as fascinating supports for heterogeneous catalysis [29,30,31]. These materials are made of the periodic stacking of positively charged di- and tri-valent metal hydroxides octahedral layers (brucite-like sheets) and negatively charged interlayer spaces where anions and water are located. Their high specific surface area and abundant hydroxyl groups facilitate the efficient anchoring of metal particles and strong metal–support interactions. Moreover, depending on the nature of the metal centers involved in an LDH, the latter can exhibit visible light absorption and, thus, improved light harvesting as photocatalysts with the potential for electronic coupling [32,33,34,35,36]. In particular, Z-scheme heterojunctions involving LDH-based materials have demonstrated remarkable photocatalytic performances, and they are employed in a wide range of photocatalytic reactions such as CO_2_ reduction, water splitting, and pollutant degradation, as very recently well-reviewed by Ding et al. [37]. Through the synergistic combination of gold metal nanoparticles and two-dimensional layered double hydroxides, we hoped to take benefits of the plasmonic behavior of gold nanocatalysts, on the one hand, and of the numerous basic sites derived from hydroxyl networks and of the potential intrinsic photocatalytic activities of the LDH, on the other hand, thus allowing us to carry photocatalytic reactions in a base-free condition [38,39,40].

In this study, we investigated the efficiency of a novel Au/Ni-Al-Zr LDH photocatalyst for the selective oxidation of glucose, used as a model substrate, into corresponding gluconic acid under standardized A.M. 1.5 G light illumination. To optimize the photocatalytic conditions, an experimental design was implemented. Moreover, the reaction was conducted using long-pass filters to elucidate the reaction mechanism, thereby offering valuable insights and guiding future developments in photocatalyst modification.

## 2. Results and Discussion

### 2.1. Material Synthesis and Characterization

**Figure 1:**
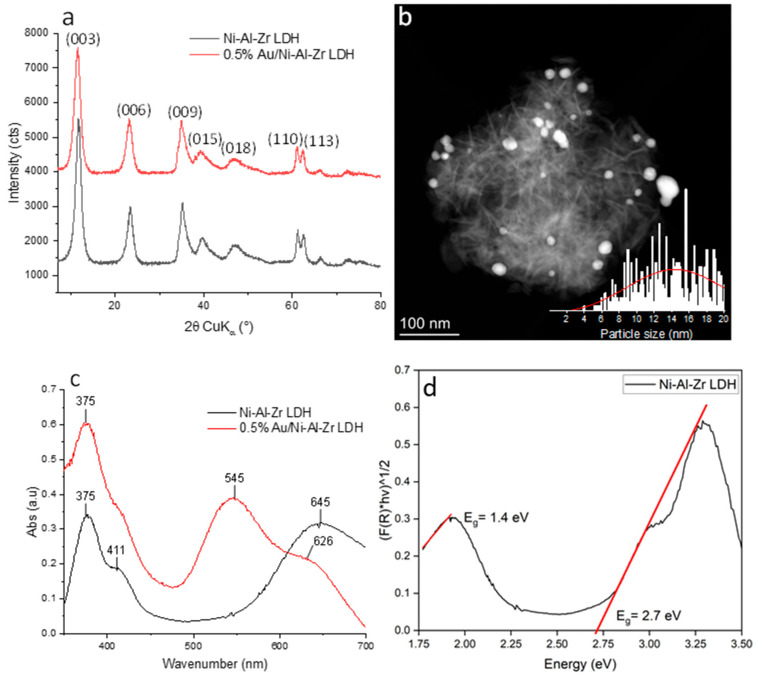
(**a**) Comparative X-ray diffractograms of the Ni-Al-Zr LDH (in black) and 0.5% Au/Ni-Al-Zr LDH (in red). (**b**) TEM micrograph in dark field of the 0.5% Au/Ni-Al-Zr LDH and the gold particle size distribution. (**c**) Comparison of the absorbance spectra of the Ni-Al-Zr LDH (in black) and the 0.5% Au/Ni-Al-Zr LDH (in red) (diffuse reflectance measured with an integration sphere). (**d**) Kubelka–Munk plot of the Ni-Al-Zr pristine material, assuming indirect allowed transition.

Among the various existing LDHs, Ni-Al-Zr LDH was selected in this study and prepared by the alkaline co-precipitation of Ni(NO_3_)_2_·6H_2_O, Al(NO_3_)_2_·6H_2_O, and Zr(NO_3_)_2_·9H_2_O salts, as previously reported [41,42]. After filtration and drying, the Au NPs were deposited on the LDH material using deposition-precipitation with a urea method [43], and the resulting Au/LDH material was fully characterized by means of X-ray diffraction (XRD), transmission electronic microscopy (TEM), UV-visible spectrometry (Figure 1), N_2_ adsorption-desorption (BET analysis, Figure S1), FT-IR spectroscopy (Figure S2), diffuse reflectance measurements represented with Kubelka–Munk function (Figure S3), thermogravimetric analysis (TGA, Figure S4), and inductively coupled plasma atomic emission spectroscopy (ICP-AES) (Table S1). The latter confirmed that the Au/Ni-Al-Zr LDH photocatalyst contained 0.49 wt.% of gold. The X-ray diffractogram (Figure 1a) shows the distinctive diffraction peaks of the layered double hydroxides based on the pristine Ni-Al-Zr LDH material, in accordance with Ni-Al-based LDH materials ([44], ICDD PDF 22-0452). Changes in the lattice parameters (Table S2) indicated a modification in the crystalline lattice of the LDH material due to the possible incorporation of gold between the inorganic layers. In the TEM micrograph showing the Au/Ni-Al-Zr LDH material (Figure 1b), we can distinctly observe the lamellar structure of the LDH, which translated into particles having a nanoscopic, urchin-like morphology. The gold nanoparticles were also visible on the dark field image, appearing as white particles that were relatively heterogeneous in size, with a wide distribution of between 6 and 20 nm. The UV-visible measurements on a dense pellet were used to determine the optical characteristics of the two LDH samples (Figure 1c).

Furthermore, the optical properties of the two layered double hydroxide (LDH) samples were systematically analyzed using the Kubelka–Munk function, which was applied to the reflectance measurements obtained from dense pellet samples (Figure 1c). This method allowed for the precise determination of the samples’ absorption characteristics. The pristine LDH powder (black curve), which exhibited a green hue, showed two prominent absorption bands at wavelengths of 375 nm and 644 nm. These bands were indicative of the electronic transitions occurring within the material, and their presence in the visible spectrum corresponded well with the observed color of the sample. Upon introducing the gold nanoparticles (Au NPs) onto the surface of the LDH, the optical fingerprint of the material changed significantly (red curve). A new broad absorption band appeared at 545 nm, which was attributed to the localized surface plasmon resonance (LSPR) of the gold nanoparticles [45]. This phenomenon occurs when the conduction electrons on the surface of Au NPs oscillate in resonance with the incident light, leading to enhanced light absorption at specific wavelengths. The emergence of this band highlighted the successful incorporation of the Au NPs into the LDH matrix, and it also highlighted their influence on the material’s optical properties. Interestingly, the presence of the Au NPs did not merely introduce a new optical feature; rather, it also altered the behavior of the existing absorption band at 644 nm. This band, which was initially observed in the pristine LDH, remained in the Au NP-decorated sample but showed signs of an interaction with the newly introduced LSPR band (red curve). The persistence and possible modification of the 644 nm band suggested that there may have been a charge transfer between the Au NPs and the LDH support. Such an interaction could enhance the electronic coupling between metal nanoparticles and the LDH, potentially leading to improved catalytic or photocatalytic properties. This charge transfer is a crucial aspect, as it indicates that the introduction of Au NPs does not simply modify the optical absorption of the material but could also impact its electronic structure and reactivity, thereby expanding the potential applications of composite materials in fields such as photocatalysis and optoelectronics. These spectroscopic measurements also allowed us to determine the optical bandgap of the pristine material using the Kubelka–Munk function (Figure 1d). For the pristine Ni-Al-Zr LDH, two large optical transitions were identified due to the partially filled d-orbitals of the Ni (d-d transition), with a bandgap at ca. 1.4 eV and charge transfer transitions from the ligand to the metal with a bandgap of ca. 2.7 eV [46]. Upon modification with the gold nanoparticles (Au/Ni-Al-Zr LDH), the emergence of the band at 2.27 eV (545 nm), attributed to surface plasmons, skewed the measurement of the bandgap; however, it appeared to be quite similar to that measured on the pristine material (Figure S3). Additionally, the TGA, BET, and FT-IR analyses (ESI) were in accordance with what is generally observed for these kinds of materials [43]. In particular, as expected, the specific BET surface area, measured by nitrogen physisorption, slightly decreased after modification with 0.5% of gold, from 160 m^2^/g for the Ni-Al-Zr structure to 150 m^2^/g for the 0.5% Au/Ni-Al-Zr-LDH material.

### 2.2. Catalyst Efficiency

#### 2.2.1. Model Reaction

**Figure 2:**
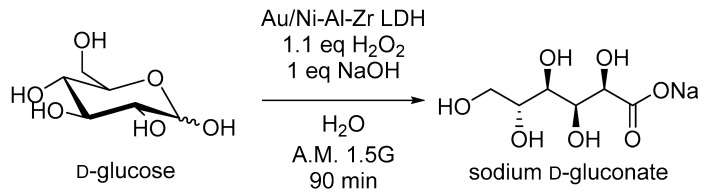
Catalytic oxidation of glucose using the 0.5% Au/Ni-Al-Zr LDH under standardized conditions.

The ability of the Au/LDH material to onset the oxidation reaction of glucose into sodium gluconate (used as the model reaction) was explored by using the photocatalytic conditions previously optimized (Figure 2) [24]. Briefly, a catalytic charge of gold (substrate/gold mol ratio: 43,000) was added to an alkaline solution of glucose (5 mol. %) in the presence of 1.1 equivalent of hydrogen peroxide as an oxidizing agent and potentially also as an electron scavenger. After 90 min of continuous illumination using standardized A.M. 1.5 G light (100 mW/cm^2^), the catalyst was filtrated, and the resulting reaction medium was freeze-dried and analyzed by NMR to determine both the conversion yield and selectivity towards sodium gluconate. As a result, this first attempt yielded only a 31% conversion despite an excellent selectivity towards sodium gluconate, thus demonstrating that transferability strongly depended on the type of photocatalyst. Consequently, we implemented an experimental plan to identify the optimal reaction conditions and estimate the effects of the main influencing parameters and interactions between them.

#### 2.2.2. Factorial Experiments

Employing a full factorial design method (FFD), four factors were precisely selected, namely, the amounts of the NaOH, H_2_O_2_, and Au/LDH photocatalyst and the time, each having two levels (high [+] and low [−]), to develop the factorial plan. Table 1 outlines the operating conditions in this study.

A total of 16 (2^4^) factorial trials were conducted (Table S3), and the conversion of glucose into gluconic acid salt was evaluated using NMR spectroscopy (Figures S7–S12). Design Expert 7.0.0 software was used to calculate the average effect and the primary interaction effects of each parameter, and the variance was analyzed using ANOVA (Table S4). All of the main effects and responses had linear relationships, as demonstrated by the linear regression equation determined from the variance analysis. The predictive model for estimating the glucose conversion yield (conversion (%)) can be expressed by the following equation:

conversion (%) = +22.50 + 14.38 × A + 5.38 × B + 16.88 × C + 2.38 × D + 8.75 × A × C + 4.75 × A × D + 4.50 × B × C

This model equation, as well as a Pareto chart (Figure S6), evidenced the importance of the factors A (m_cat_) and C (m_NaOH_) on the conversion yield of glucose. The amount of NaOH was the first and the most sensitive variable affecting the yield of conversion of glucose. This first result was interesting and counterintuitive given the basicity provided by the LDH support. This effect was positive (+16.88), meaning that the increase in the amount of NaOH increased the yield of the glucose conversion. Sodium hydroxide played a pivotal role as an alkaline agent in the process. It governed the catalytic activity of the gold nanoparticles (Au NPs) and allowed for maintaining the ideal surface conditions to avoid poisoning the catalyst, in particular, when the pH was acidic, while promoting the chemisorption of glucose by its hydrated form [47]. This result endorsed that, contrary to what one could expect, the intrinsic basicity of the LDH material was, unfortunately, not strong enough to initiate the photocatalytic process.

**Figure 3:**
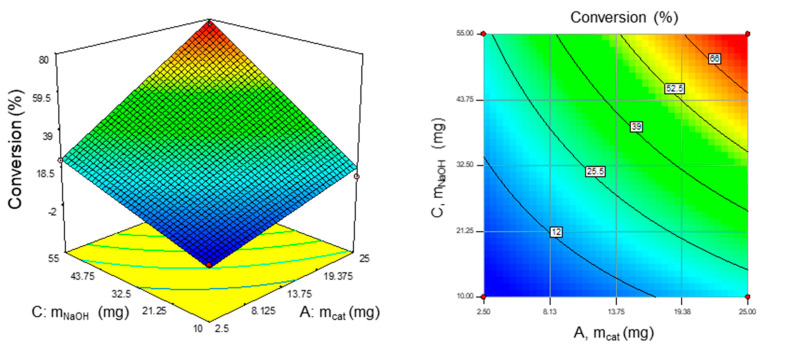
Three-dimensional response surface plot (**left**) and corresponding contour plot (**right**) of the significant AC interaction at VH_2_O_2_ = 600 µL and time = 90 min with the conversion yield.

The quantity of the catalyst was the second most important factor, having also a positive effect (+14.09). Lastly, our results underlined that the reaction time and H_2_O_2_ quantity were not influencing factors. The most important interaction worth mentioning was that occurring between the weight of the NaOH and the catalyst (+8.75). Examining surface plots as a function of two factors while maintaining constant values of others is an effective method for determining the relationship between a reaction condition and a conversion yield as a response. Figure 3 provides three-dimensional and contour plots regarding the effect of the most significant two-factor interactions (AC, weights of the NaOH and catalyst) on the yield of glucose conversion, confirming the positive effect of both factors.

Utilizing Expert Design 7.0.0 software, a numerical optimization technique was employed to determine the optimal conditions for glucose conversion, as displayed in Table 2.

By experimentally applying the conditions determined by the numerical model, a noticeable gain in conversion yield to 87% was obtained. This result corroborated well with the predicted conversion yield value determined by the FFD method. Indeed, this latter value was the best result obtained with this catalyst, which allowed us to further confirm the accuracy and validity of the herein-proposed predictive model. Moreover, in comparison with the recently published results summarized in Table 3, this material demonstrated a remarkable performance, achieving one of the highest-reported glucose conversion rates and exceptional selectivity toward gluconic acid salt within just 1.5 h at room temperature. Nevertheless, it did not surpass the results we previously achieved with an Au/CeO_2_ material, which enabled quantitative and selective glucose conversion in only 10 min under standardized illumination [24].

### 2.3. Photocatalytic Behavior and Possible Mechanism

**Figure 4:**
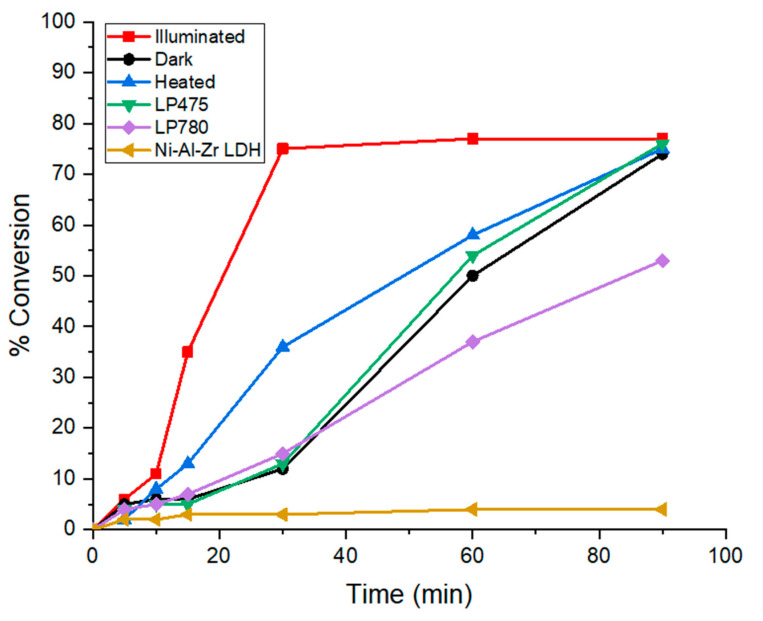
Kinetic comparison of glucose oxidation under standardized A.M. 1.5 G illumination (red), dark conditions (black), or heating to 35 °C (blue). In addition, long-pass filters at 475 nm (green) and 780 nm (purple) were used for incident light spectrum selection. As indicated in yellow, a reaction was performed using the LDH alone.

Simultaneously to the factorial experiments, we observed a noticeable variation when standardized illumination or dark conditions were used at room temperature. To decipher if a light-induced mechanism was herein involved, the reaction was deliberately conducted in the dark (Figure 4, black curve). Compared to the darkness, it was clear that in the presence of light (Figure 4, red curve), a much faster reaction was observed, reaching a maximum of 77% conversion yield within only 30 min vs. 74% conversion yield after 90 min in darkness. This first comparative result stressed the contribution of incident light in accelerating the kinetics of glucose oxidation, and therefore, it emphasized the photocatalytic nature of the reaction. Nevertheless, after 90 min of continuous illumination, the reaction medium temperature reached 35 °C. To confirm the photocatalytic hypothesis and also to rule out any heat-induced activation for this reaction, a similar reaction was conducted in the dark at a temperature of 35 °C. As shown in Figure 4 (blue curve), the kinetic enhancement by temperature remained marginal, thus excluding any thermal effect.

The involvement of photon-induced free carriers in glucose oxidation demonstrated that the following two potential mechanisms could be proposed: (1) a charge transfer reaction from the LDH bandgap excitation into the gold nanoparticles, and (2) a gold plasmonic-induced charge transfer reaction to the LDH.

To identify the main activation path, we introduced long-pass filters with two different cutoff wavelengths between the light source and the photocatalytic reactor (475 nm and 780 nm). The selection of these filters was guided by the optical characteristics of the photocatalyst, i.e., Ni-Al-Zr LDH has maximum absorptions at 375 nm and 644 nm, whereas the LSPR of gold nanoparticles is located at ca. 550 nm. Thus, the 475 nm filter principally annihilated the high energy photons excitation of the LDH band at 375 nm while leaving the possibility of LSPR excitation. In contrast, the 780 nm long-pass filter hampered both the gold and LDH excitation. Interestingly, when utilizing an LP780 nm filter (Figure 4, purple curve), there appeared to be no discernible impact of the filtered light on the kinetics of the glucose conversion compared to the darkness experiment. A slight slowing down, particularly at longer times such as 90 min, was even observed, likely due to fluctuations related to the ambient temperature and/or the larger standard deviations. This confirmed that the near-infrared radiation of the Xe-lamp-filtered A.M. 1.5 G condition was not responsible for the photo-induced activation. This was in good agreement with the dark temperature test at 35 °C. Interestingly, it was observed that when employing the LP475 nm filter (Figure 4, green curve), the glucose conversion rate was very similar to that obtained in darkness. This result led us to the conclusion that the photocatalytic activity stemmed from the high-energy photon bandgap excitation in UV and that the Au NPs excitation was not responsible for the photon-induced oxidation of the glucose. Further, the necessity of the gold deposition on the LDH material was undoubtedly highlighted by the experience involving the support material alone under light, yielding a 4% conversion rate after 90 min (yellow curve).

**Figure 5:**
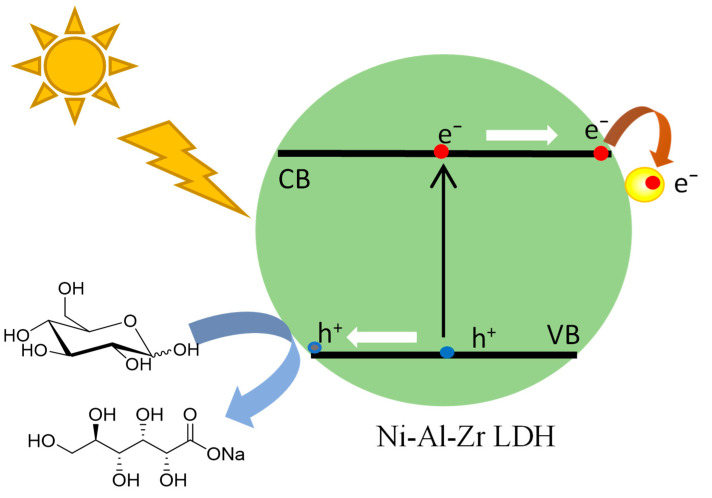
Proposed mechanism for the selective glucose oxidation using Au/Ni-Al-Zr LDH under solar-simulated illumination.

In conclusion, a charge-driven transfer process starting from the excitation of the LDH to the supported gold nanoparticles was evidenced by this bundle of results (Figure 5). As evidenced by the reaction made without H_2_O_2_, which resulted in no reaction, the latter served as an electron scavenger after the injection of electrons from the conduction band of the LDH into the gold. This electron-scavenging process enhanced the charge separation lifetime, consistent with previous observations in the literature [24,47,48,49], thereby preventing the undesired rapid radiative recombination processes. This electron transfer resulted in the formation of ·OH oxidant radicals and HO⁻ anions, which also prevented the poisoning of the gold catalyst by the as obtained gluconic acid. This mechanism knowledge will help to design new materials with tunable bandgaps of the support, allowing for better harvesting of the light and, thus, a better efficiency for the selective transformation of free carbohydrates.

## 3. Materials and Methods

### 3.1. General

The solvents and chemical reagents, such as the nickel nitrate (Ni(NO_3_)_2_·6H_2_O), aluminum nitrate (Al(NO_3_)_2_·6H_2_O), zirconium nitrate (Zr(NO_3_)_2_·9H_2_O), sodium carbonate, tetrachloroauric acid (HAuCl_4_·3H_2_O), sodium hydroxide, urea, and glucose, were purchased from Merck-Sigma-Aldrich (St Quentin Fallavier, France) or Thermofischer Scientific (Illkirch, France), and used as received.

### 3.2. Synthesis of the Ni-Al-Zr LDH

The Ni-Al-Zr LDH was prepared by a co-precipitation method, as previously reported [41,42]. A solution containing the Ni(NO_3_)_2_·6H_2_O, Al(NO_3_)_2_·6H_2_O, and Zr(NO_3_)_2_·9H_2_O in 100 mL of distilled water was slowly dropped under magnetic stirring into a 0.5 M Na_2_CO_3_ aqueous solution at a rate of 0.1 mL/min. The pH was raised to 10 by adding 2 M NaOH. The precipitate was separated by centrifugation and washed several times with distilled water until the excess nitrates and carbonates were completely removed. The samples were then dried in an oven at 80 °C for 24 h and, finally, ground into fine powders.

### 3.3. Preparation of the Au/Ni-Al-Zr LDH

The 0.5% Au/Ni-Al-Zr LDH catalyst was prepared by deposition precipitation with urea, as described elsewhere [43]. The Ni-Al-Zr LDH support, prepared by co-precipitation, was dispersed in 200 mL of distilled water under agitation until the temperature reached 80 °C. Subsequently, 0.5 wt. % of gold from HAuCl_4_·3H_2_O (at a concentration of 10 g/L) and urea were added to the mixture, and the mixture was stirred for 16 h.

### 3.4. Catalytic Test

In the optimized procedure, 1.38 mmol of sugar and 55 mg of NaOH (1 equivalent) were dissolved in 6 mL of water within a glass tube. Subsequently, 25 mg of catalyst, consisting of Au/NiAl_2_Zr_4.5_ LDH (0.5 wt. %) and 200 µL of 30% aq. H_2_O_2_, were added to the solution, and then the reaction mixture was stirred at room temperature under standardized illumination (A.M. 1.5 G) for 90 min. After the reaction, the catalyst was separated by filtration through a nylon membrane (0.25 μm). The resulting filtrate was freeze-dried, and the crude product was characterized by ^1^H NMR in D_2_O. The area of the H_2_ peak of the synthesized gluconate (δ = 4.14 ppm, d, *J* = 3.7 Hz) was compared to the area of the H1 peaks of the residual glucose (H1α: δ = 5.24 ppm, d, *J* = 3.7 Hz and H1β: δ = 4.65 ppm, d, *J* = 7.9 Hz) to calculate a conversion rate according to the following formula:

$$\%\;Conv.=\frac{A(H2gluconate)}{A(H1\alpha glucose)+A(H1\beta glucose)+A(H2gluconate)}\times 100$$

## 4. Conclusions

In conclusion, this study highlights the important role of the charge transfer induced by the excitation of the matrix of layered double hydroxides (LDHs) to support gold nanoparticles. This process constitutes a fundamental mechanism for enhancing the photocatalytic properties of the studied materials. The successful integration of gold nanoparticles within the Ni-Al-Zr LDH matrix not only preserved the base crystalline structure but also facilitated efficient electron transfer, which is essential for the activation and selective conversion of free carbohydrates.

To further elucidate this understanding, a factorial design with four parameters was employed, enabling the optimization of the reaction conditions and the systematic exploration of the influence of each factor on overall process efficiency. This experimental design was instrumental in identifying the optimal conditions, minimizing the number of trials required while maximizing the information obtained on the parameter interactions. Through this methodical approach, specific parameter ranges where photocatalytic yield was maximized, particularly in terms of the adjusted bandgap of the LDH, could be targeted.

The insights gained into this charge transfer mechanism open promising avenues for the design of new materials with finely tunable optoelectronic properties. By manipulating the bandgaps of LDH supports, it becomes feasible to optimize solar light absorption, thereby expanding the usable light spectrum for photocatalytic reactions. This enhanced light capture translates into substantial improvements in the efficiency of selective chemical transformations, particularly in the conversion of free carbohydrates, a major area of interest in green chemistry and renewable energy.

In summary, this study not only demonstrates enhanced photocatalytic capabilities of Au/LDH composites but also provides a robust methodology for the rational design of advanced materials. These advancements could have significant implications in the development of more sustainable chemical processes, fully leveraging solar light for industrial and environmental applications.

## Figures and Tables

**Table 1:** Factors and levels studied.

Factors	Level		
	Low (−)	Center (0)	High (+)
A—Catalyst mass (mg)	2.5	13.75	25
B—H_2_O_2_ volume (µL)	200	400	600
C—NaOH mass (mg)	10	32	55
D—Time (min)	30	60	90

**Table 2:** Predicted (Pred.) and experimental (Exp.) results obtained for the optimal operating conditions determined using a factorial plan.

	m_cat_ (mg)	V(H_2_O_2_) (µL)	m_NaOH_ (mg)	Time (min)	Conv. (%)
Pred.	29.7	519.13	64.92	89.89	100
Exp.	30	520	65	90	87

**Table 3:** Photocatalytic performance of various catalysts for glucose conversion under visible-light irradiation.

Catalyst	Illumination	Conditions	Glucose Conversion	Selectivity	Ref.
4.7Mg–CN/CS	500 W Xe short arc lamp with a cut-off filter of λ > 380 nm	50 °C/2 h	97%	71% lactic acid	[13]
Zn_0.6_Cd_0.4_S	300 W Xenon lamp	25 °C	~90%	~87% lactic acid	[14]
0.2%Pt/TiO_2_	300 W Xenon lamp	15 °C/4 h	35.9%	78.6% arabinose and 21.0% erythrose	[11]
0.2%Rh/TiO_2_	300 W Xenon lamp	15 °C/4 h	47.0%	74.7% arabinose and 20.6% erythrose	[11]
0.2%Ni/TiO_2_	300 W Xenon lamp	15 °C/4 h	27.7%	85.9% arabinose and 7.5% erythrose	[11]
TiO_2_	300 W Xenon lamp	15 °C/4 h	42%	7% gluconic acid and 93% C_2_–C_5_ compounds	[11]
Au/CeO_2_	Simulated sunlight (A.M. 1.5 G)	25 °C/10 min	>99%	>95% sodium gluconate	[24]
0.5% Au/Ni-Al-Zr LDH	Simulated sunlight (A.M. 1.5 G)	25 °C/1.5 h	87%	>95% sodium gluconate	This work

## Data Availability

The original contributions presented in the study are included in the article and the Supplementary Materials. Further inquiries can be directed to the corresponding author.

## Supplementary Materials

The following supporting information can be downloaded at: https://www.mdpi.com/article/10.3390/molecules30010013/s1, instrument specifications, material characterization (BET analysis, FT-IR spectroscopy, ICP-AES, TGA, and XRD), factorial experiments, and the ^1^H NMR spectra of the crudes.
