# Supplementary material for: Exploring the Photocatalytic Efficiency of Gold Nanoparticles Deposited on Ni-Al-Zr-Layered Double Hydroxides for Selective Glucose Oxidation
Source: Molecules. 2024 Dec 24;30(1):13. doi: 10.3390/molecules30010013 (PMC11721991; doi:10.3390/molecules30010013)
Supplement: Supplementary file 1 [file molecules-30-00013-s001.zip › molecules-3372391-supplementary.pdf]

# Supporting Information

# Exploring the Photocatalytic Efficiency of Gold Nanoparticles Deposited on Ni-Al-Zr-Layered Double Hydroxides for Selective Glucose Oxidation

**Nihel Dib [1,2,3], Frédéric Sauvage [4], Lucie Quéhon [4], Khadidja Khaldi [5], Sumeya Bedrane [1], José Juan Calvino [2,3], Redouane Bachir [1], Ginesa Blanco [2,3] and Gwladys Pourceau [4,6,*]**

[1] Laboratory of Catalysis and Synthesis in Organic Chemistry (LCSCO), University of Tlemcen, BP 119, 13000 Tlemcen, Algeria
[2] Departamento de Ciencia de los Materiales, Ingeniería Metalúrgica y Química Inorgánica, Facultad de Ciencias, Universidad de Cádiz, Campus Río San Pedro, E-11510 Puerto Real, Cádiz, Spain
[3] Instituto Universitario de Investigación en Microscopía Electrónica y Materiales (IMEYMAT), Universidad de Cádiz,Campus Río San Pedro, E-11510 Puerto Real, Cádiz, Spain
[4] Laboratoire de Réactivité et Chimie des Solides (LRCS), UMR CNRS 7314, Université de Picardie Jules Verne, Hub de l'énergie, 15 rue Baudelocque, FR-80000 Amiens, France
[5] Centre de Recherche Scientifique et Technique en Analyses Physico-Chimiques CRAPC, BP 384, 42004, Tipaza, Algeria
[6] Laboratoire de Glycochimie et des Agroressources d'Amiens (LG2A) UR 7378, Université de Picardie Jules Verne, FR-80000 Amiens, France
* Correspondence: gwladys.pourceau@u-picardie.fr

## Table of contents

1. Instruments specifications ........ 2
2. Materials characterizations ........ 3
   a. $N_2$ adsorption-desorption (BET analysis) ........ 3
   b. FT-IR spectroscopy ........ 4
   c. Kubelka-Munk plots derived from diffuse reflectance measurements ........ 5
   d. Metal loading measured by ICP-AES ........ 5
   e. Thermogravimetric analysis ........ 5
   f. X-ray diffraction ........ 6
3. Factorial experiments ........ 6
4. NMR spectra ........ 11
5. References ........ 17

# 1. Instruments specifications

NMR analyses were performed on a spectrometer Bruker operating at 400 MHz for $^{1}H$ and 100 MHz for $^{13}C$ NMR samples were prepared in $D_2O$. Assignments of $^{1}H$ and $^{13}C$ signals were performed using correlated spectroscopy (COSY) and heteronuclear single quantum correlation (HSQC).

X-ray powder diffractograms (XRD) are determined in the range of $2\theta = 10\text{-}80°$ using a Rigaku Mini flex 600 diffractometer with Ni-filtered Cu K$\alpha$ radiation ($\lambda$ = 1.541874 Å) with a scanning speed of 2 steps per second and a step of 0.02°.

High-angle annular dark-field images (HAADF-STEM) were collected with a FEI TALOS F200X microscope. The samples for TEM were prepared as follow: the catalyst was suspended in EtOH, a drop was deposited on a Cu grid with holey carbon support membrane and the grid was let dry 15 h. Gold particle size distribution was determined by counting 100 particles from multiple separate catalyst particles.

The optical properties of the samples were measured by UV–Visible spectroscopy (200–800 nm) using a Cary Series UV-Visible-NIR spectrophotometer equipped with a set of diffuse reflectance accessories (integration sphere) to collect only the diffusion of the reflected light.

After dissolving the catalyst into aqua regia ($HCl/HNO_3$ 3:1), the metal content of the catalyst was determined by inductively coupled plasma atomic emission spectroscopy (ICP-AES) using Thermo Elemental Iris intrepid equipment.

A full factorial design was used to investigate the photocatalytic efficiency of Au/Ni-Al-Zr LDH in glucose oxidation. This technique enables the simultaneous evaluation of individual factors, their interactions, and their impact on the photocatalytic process. Consequently, it allows the optimization of operating conditions. The evaluation of these parameters was achieved through analysis of variance (ANOVA) using Design Expert®7 software.

# 2. Materials characterizations

## a. $N_2$ adsorption-desorption (BET analysis)

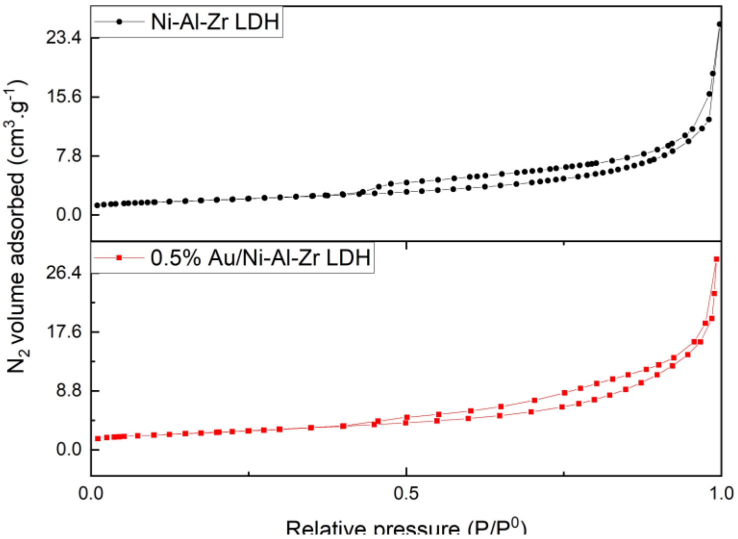


Figure S1: $N_2$ adsorption–desorption isotherm at 77K of Ni-Al-Zr LDH (black), 0.5%Au/Ni-Al-Zr LDH (red).

The texture properties of the porous LDH was determined by physisorption isotherms using nitrogen ($N_2$) at its liquefaction temperature (77K). Prior to physisorption measurements, the solid surface samples were outgassed at 200 °C for 2 hours under vacuum to eliminate all traces of water, carbonates, and gas residues.

The nitrogen adsorption-desorption isotherms of Ni-AL-Zr LDH (in black) and 0.5%Au/Ni-Al-Zr LDH (in red) are shown in Figure S1. They are similar to those recorded for the lamellar double hydroxide compound [1,2]. The adsorption/desorption isotherm is class IV [3,4] indicating mesoporosity of the two materials. The hysteresis is of H3 type suggesting a slot-like mesopore model where the mesoporosity is created by the aggregation of particles.

## b. FT-IR spectroscopy

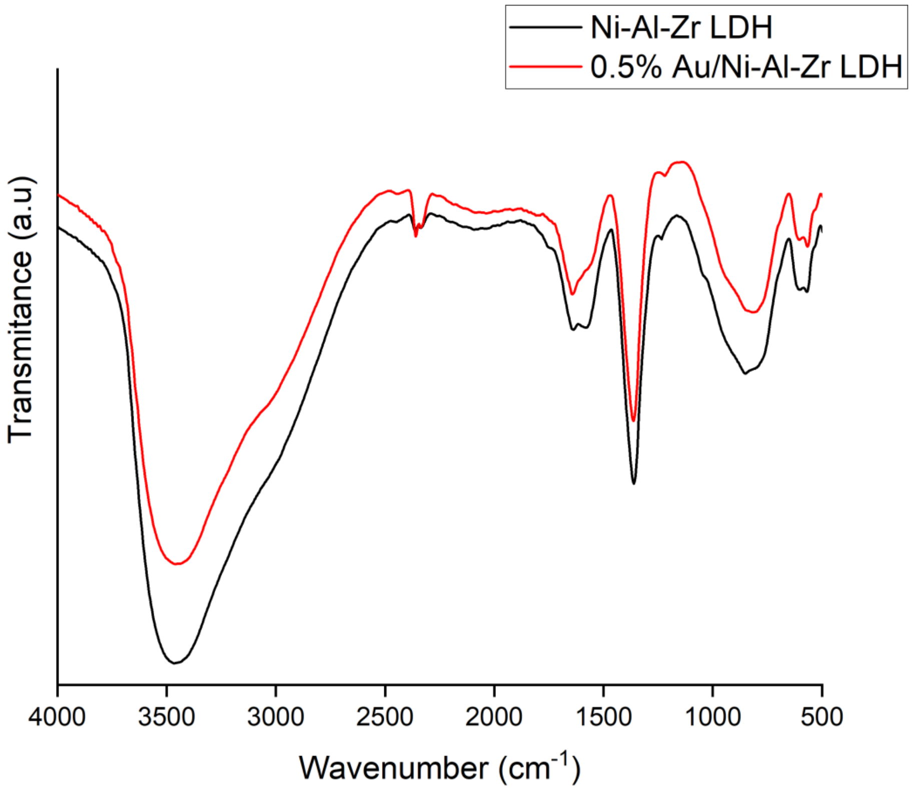


Figure S2: FT-IR spectra of Ni-Al-Zr LDH (black) and 0.5%Au/Ni-Al-Zr LDH (red).

Figure S2 exhibits IR spectra of pristine Ni-Al-Zr LDH (black curve) and 0.5%Au/Ni-Al-Zr LDH (red curve). Both samples exhibited a broad intense band around 3460 $cm^{-1}$, attributed to the OH stretching mode of the layer's hydroxyl groups and the interlayer's $H_2O$ molecules [5]. The band in the 2300–2350 $cm^{-1}$ range is due to $CO_2$ [6]. A medium-intensity absorption band around 1600 $cm^{-1}$ corresponds to the deformation (νHOH bending) mode of $H_2O$ molecules. A sharp intense band at 1360 $cm^{-1}$ represents the $CO_3^{2-}$ ion stretching vibration [7]. The bands observed below 1000 $cm^{-1}$ are generally attributed to the vibrations of M–O, M–O–M, and O–M–O bonds in the brucite-like lattice [8,9].

## c. Kubelka-Munk plots derived from diffuse reflectance measurements

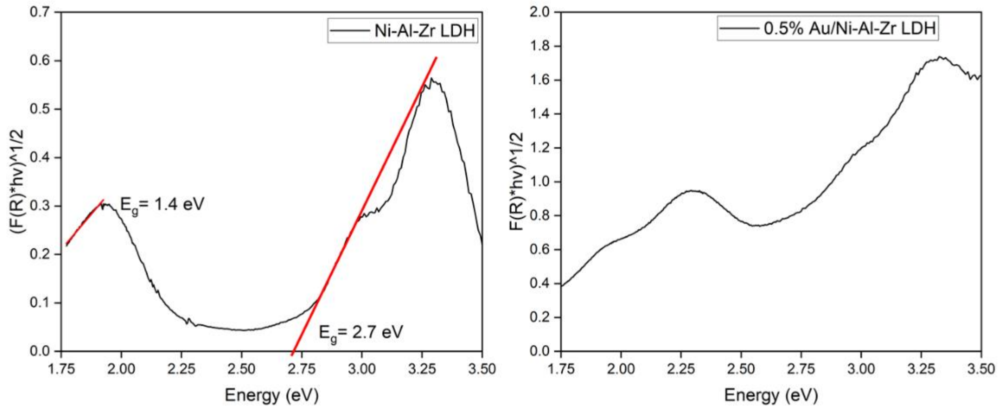


Figure S3: Diffuse reflectance measurements represented with Kubelka-Munk function for Ni-Al-Zr LDH (left, yet shown in the main article) and 0.5%Au/Ni-Al-Zr LDH (right).

## d. Metal loading measured by ICP-AES

Table S1: Metal loading measured by ICP-AES.

| Samples | Au (wt.%) | Ni (wt.%) | Al (wt.%) | Zr (wt.%) |
|---|---|---|---|---|
| Ni-Al-Zr LDH | / | 35.0±0.5 | 6.60±0.20 | 2.20±0.04 |
| 0.5%Au/Ni-Al-Zr LDH | 0.49±0.01 | 39.0±0.4 | 6.80±0.10 | 2.20±0.01 |

## e. Thermogravimetric analysis

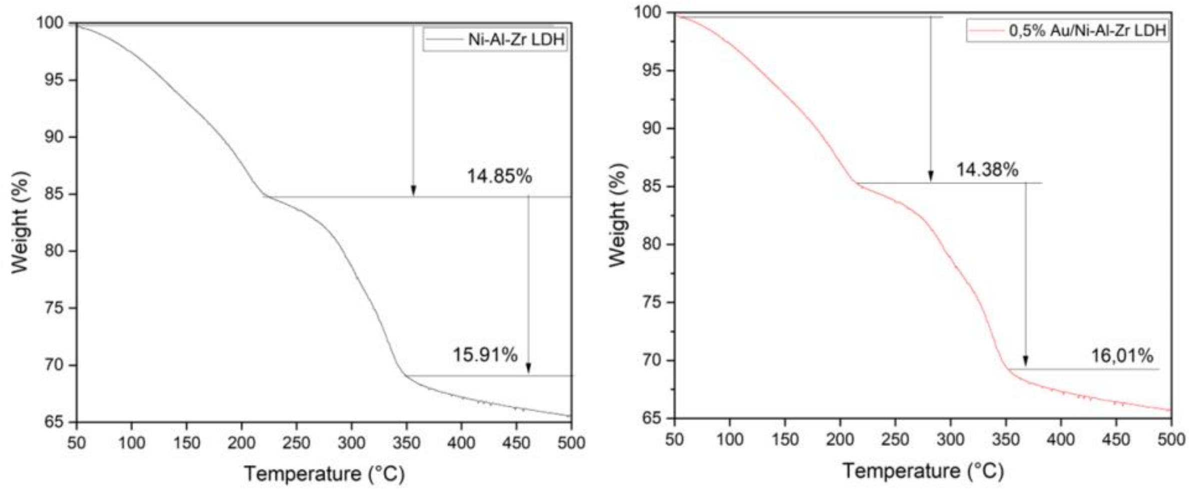


Figure S4: Thermogravimetric curve obtained for Ni-Al-Zr LDH (black) and 0.5% Au/Ni-Al-Zr LDH under air (red).

The thermogravimetric analysis (TGA) was employed to investigate the characteristics of Ni-Al-Zr LDH and Ni-Al-Zr LDH containing 0.5% gold, over a temperature range of 25 to 500 °C in air atmosphere. The corresponding TG curves are depicted in Figure S4. The results reveal overlapping mass decomposition profiles for Ni-Al-Zr LDH and 0.5% Au/Ni-Al-Zr LDH, with two main stages of mass loss. The first mass loss, approximately 15% at 220 °C, is attributed to the physical desorption of surface water molecules and loss of structural water molecules sitting in the interlayers of the LDH. Between 220 and 350 °C, a mass loss of about 16% is observed, resulting from a combination of decarbonation and dehydroxylation of the material [10,11] This decomposition occurs in the same temperature range for both Ni-Al-Zr LDH and its 0.5% gold derivative. It is noteworthy that the addition of 0.5% gold did not significantly impact the thermal properties of the LDH.

### f. X-ray diffraction

Lattice cell parameters (Table S2) were calculated based on the X-ray diffractograms depicted in the main article (Fig. 1a) and in accordance with the 22-0452 XRD PDF card provided for Ni-Al LDH in literature [12]. Upon deposition of gold onto the LDH surface (red curve), it's notable how the peaks shift towards smaller 2θ angles, revealing interactions between gold atoms and LDH. The adding 0.5% gold to Ni-Al-Zr LDH resulted in a decrease in the lattice parameter a from 3.02 Å to 2.98 Å, while the lattice parameter c increased from 22.74 Å to 22.78 Å. This suggests a contraction of the crystal lattice along the a direction and a slight expansion along the c direction.

Table S2: Lattice cell parameters of the two LDH samples.

| **Lattice parameter** | **$d_{(110)}$ (Å)** | **a (Å)** | **$d_{(003)}$ (Å)** | **c (Å)** |
|---|---|---|---|---|
| **Ni-Al-Zr LDH** | 1.51 | 2.14 | 7.58 | 22.74 |
| **0.5% Au/Ni-Al-Zr LDH** | 1.49 | 2.11 | 7.59 | 22.78 |

## 3. Factorial experiments

Employing a full factorial experimental design method, four factors were chosen, each having two levels (high [+] and low [−]), to develop the factorial plan as shown in Table 1 of the main manuscript. A total of 16 ($2^4$) factorial trials were conducted as shown in Table S3, displaying the results of experimental measurements for the glucose conversion.

Table S3: Design matrix for $2^4$ full-factorial design and corresponding experimental responses ($m_{glucose}$ = 250 mg, $V_{H2O}$ = 6 mL, A.M. 1.5 G illumination).

| Trial n° | Catalyst weight (g) | $H_2O_2$ volume (µL) | NaOH weight (mg) | Reaction time (min) | Conversion yield (%) |
|---|---|---|---|---|---|
| 1 | 25 | 200 | 10 | 30 | 6 |
| 2 | 2.5 | 200 | 10 | 30 | 0 |
| 3 | 25 | 600 | 10 | 30 | 12 |
| 4 | 2.5 | 600 | 10 | 30 | 0 |
| 5 | 25 | 200 | 55 | 30 | 27 |
| 6 | 2.5 | 200 | 55 | 30 | 18 |
| 7 | 25 | 600 | 55 | 30 | 74 |
| 8 | 2.5 | 600 | 55 | 30 | 24 |
| 9 | 25 | 200 | 10 | 90 | 13 |
| 10 | 2.5 | 200 | 10 | 90 | 0 |
| 11 | 25 | 600 | 10 | 90 | 14 |
| 12 | 2.5 | 600 | 10 | 90 | 0 |
| 13 | 25 | 200 | 55 | 90 | 73 |
| 14 | 2.5 | 200 | 55 | 90 | 0 |
| 15 | 25 | 600 | 55 | 90 | 76 |
| 16 | 2.5 | 60 | 55 | 90 | 23 |

Design Expert software was used to calculate the average effect and the primary interaction effects of the conversion of glucose into gluconic acid salt. The results of the analysis of variance (ANOVA) are presented in Table S4.

Table S4: Analysis of variance table [Partial sum of squares - Type III of conversion response].

| Source | Sum of Squares | df | Mean Square | F Value | P-value Prob > F | Conclusion |
|---|---|---|---|---|---|---|
| Model | 10325.00 | 7 | 1475.00 | 13.74 | 0.0007 | Significant |
| A-$m_{cat}$ | 3306.25 | 1 | 3306.25 | 30.79 | 0.0005 | Significant |
| B-$V_{H2O2}$ | 462.25 | 1 | 462.25 | 4.31 | 0.0717 | Significant |
| C-$m_{NaOH}$ | 4556.25 | 1 | 4556.25 | 42.43 | 0.0002 | Significant |
| D-Time | 90.25 | 1 | 90.25 | 0.84 | 0.3861 | Not significant |
| AC | 1225.00 | 1 | 1225.00 | 11.41 | 0.0097 | Significant |
| AD | 361.00 | 1 | 361.00 | 3.36 | 0.1041 | Not significant |
| BC | 324.00 | 1 | 325.00 | 3.02 | 0.1206 | Not significant |
| Residual | 859.00 | 8 | 107.38 | | | |
| Cor Total | 11184.00 | 15 | | | | |

The model F-value is 13.74 for the conversion response. It should be noted that "Prob > F" values below 0.05 indicate that the model terms are significant. Conversely, a "P-value" greater than 0.1 suggests the non-significance of the model terms. Notably, the extremely low p-value (< 0.0001) underscores the rarity of the "model F-value" occurring due to experimental noise, with only a 0.01% chance.

The main factors A ($m_{cat}$) and C ($m_{NaOH}$) are significant. Values between 0.05 and 0.10, such as AC are considered slightly significant. The conversion response model exhibits high determination values: $R^2$ = 0.9232, Adj-$R^2$ = 0.8560 and pred $R^2$ = 0.6928.

The obtained polynomial equation through multiple regression analysis in coded units provides a means to relate the rate of conversion reaction of glucose to gluconic acid salt to the investigated factors. All main effects and responses have a linear relationship, as demonstrated by the regression equation that was produced from the variance analysis. The predictive models for estimating the conversion of glucose (Conversion (%) can be expressed by the following equation (yet shown in the main article):

$$\text{Conversion (\%)} = + 22.50 + 14.38 \times A + 5.38 \times B + 16.88 \times C + 2.38 \times D + 8.75 \times A \times C + 4.75 \times A \times D + 4.50 \times B \times C$$

Figure S5 illustrates how well this model explains the experimental range under investigation by comparing the graphical representation of actual values vs. predicted values. The previous results are verified by a Pareto chart (Figure S6) displaying the t-values of the effects, which are proportional to their degree

of significance. Values of the effects are calculated by the formula and compared with reference lines: standard t-Value Limit = 2.306 for conversion model. On the other hand, the Bonferroni limit = 4.122 in the conversion model is more rigorous. Effects surpassing the Bonferroni limit are undoubtedly significant to highly important, whereas those exceeding the standard t-value limit might range from potentially significant to moderately important [13].

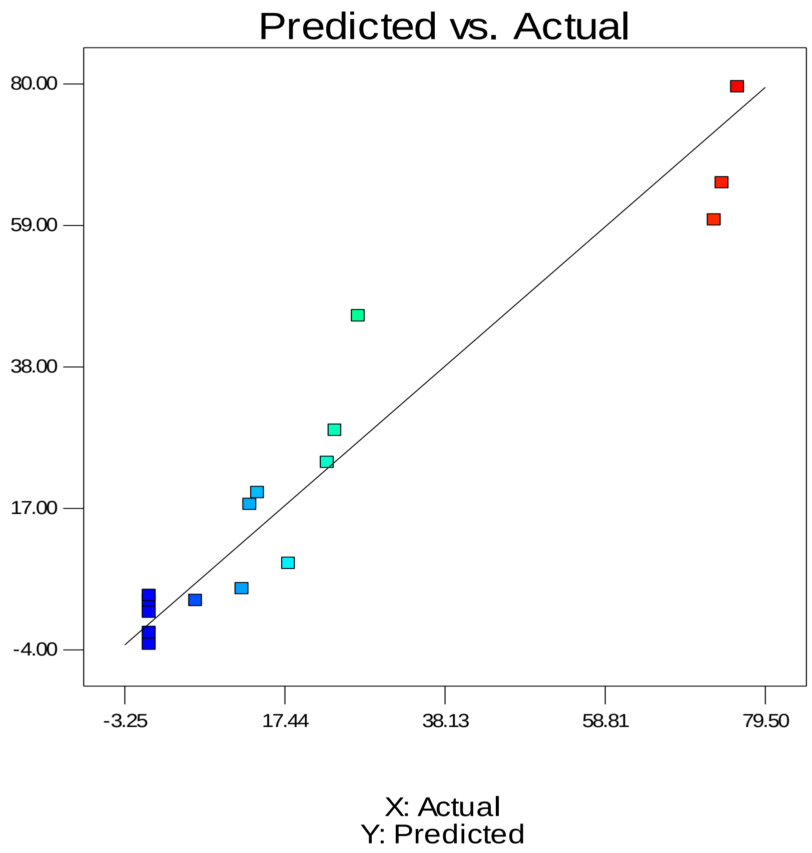


Figure S5: Plot of actual values vs. predicted values.

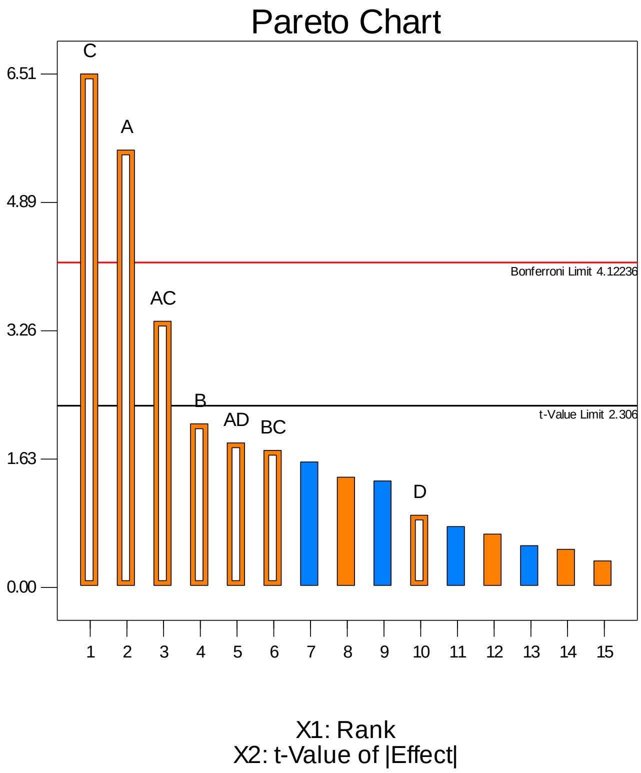


Figure S6: Pareto chart of effects.

# 4. NMR spectra

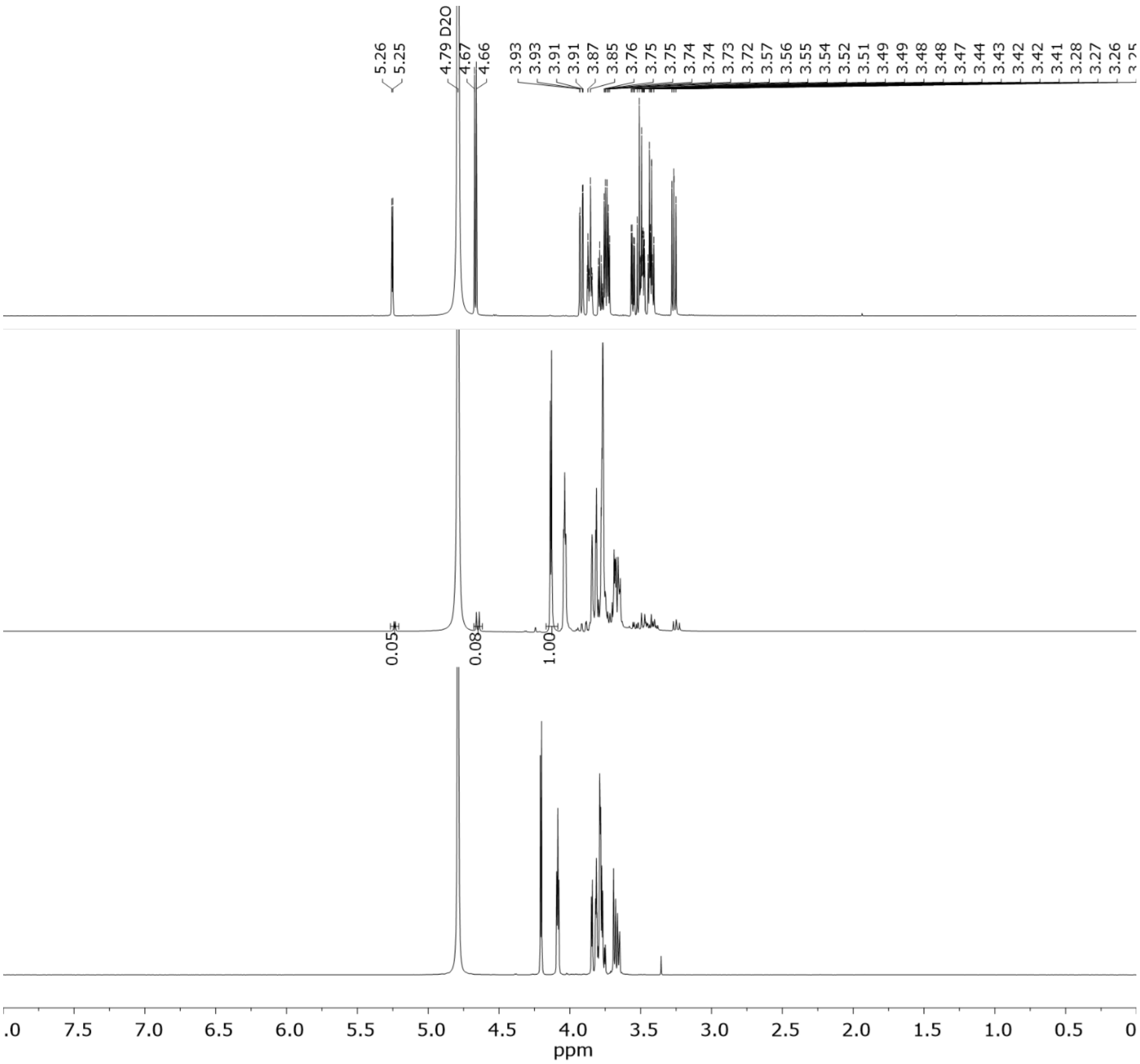


<u>Figure S7</u>: $^1$H NMR spectra (in $D_2O$) obtained for glucose (top), experimental crude obtained using the conditions proposed by the factorial plan (middle, conditions of Table 2) and commercial gluconic acid (bottom).

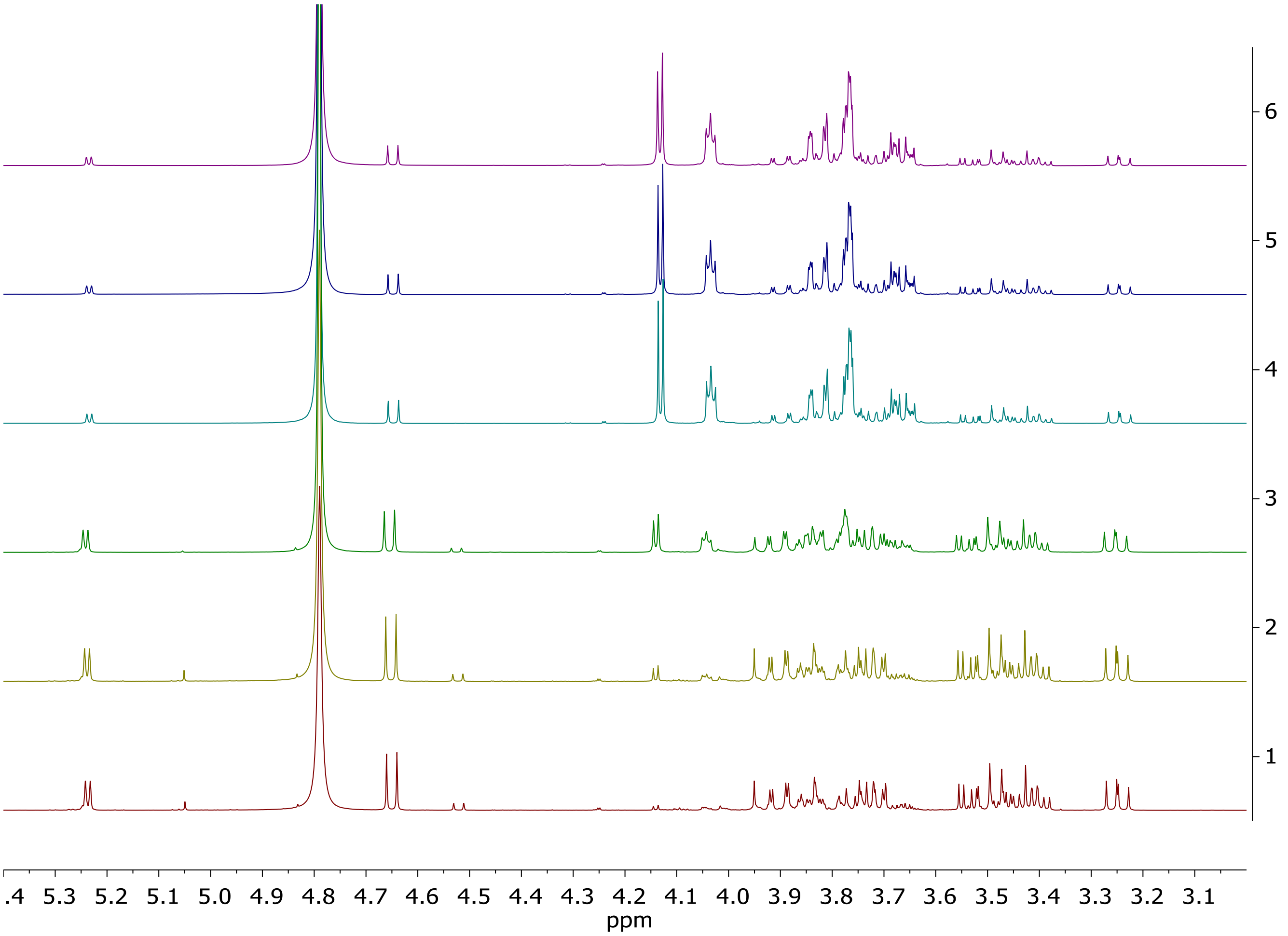


<u>Figure S8</u>: $^1$H NMR spectra (in $D_2O$) of the crude obtained under A.M. 1.5 G illumination at several times (red-1: 5 min, yellow-2:10 min, green-3: 15 min, cyan-4: 30 min, blue-5: 60 min, purple-6: 90 min). *Cond.:* $m_{glucose}$ = 250 mg, $m_{cat}$ = 25 mg, $m_{NaOH}$ = 55 mg, $V_{H2O2}$ = 200 µL, $V_{H2O}$ = 6 mL.

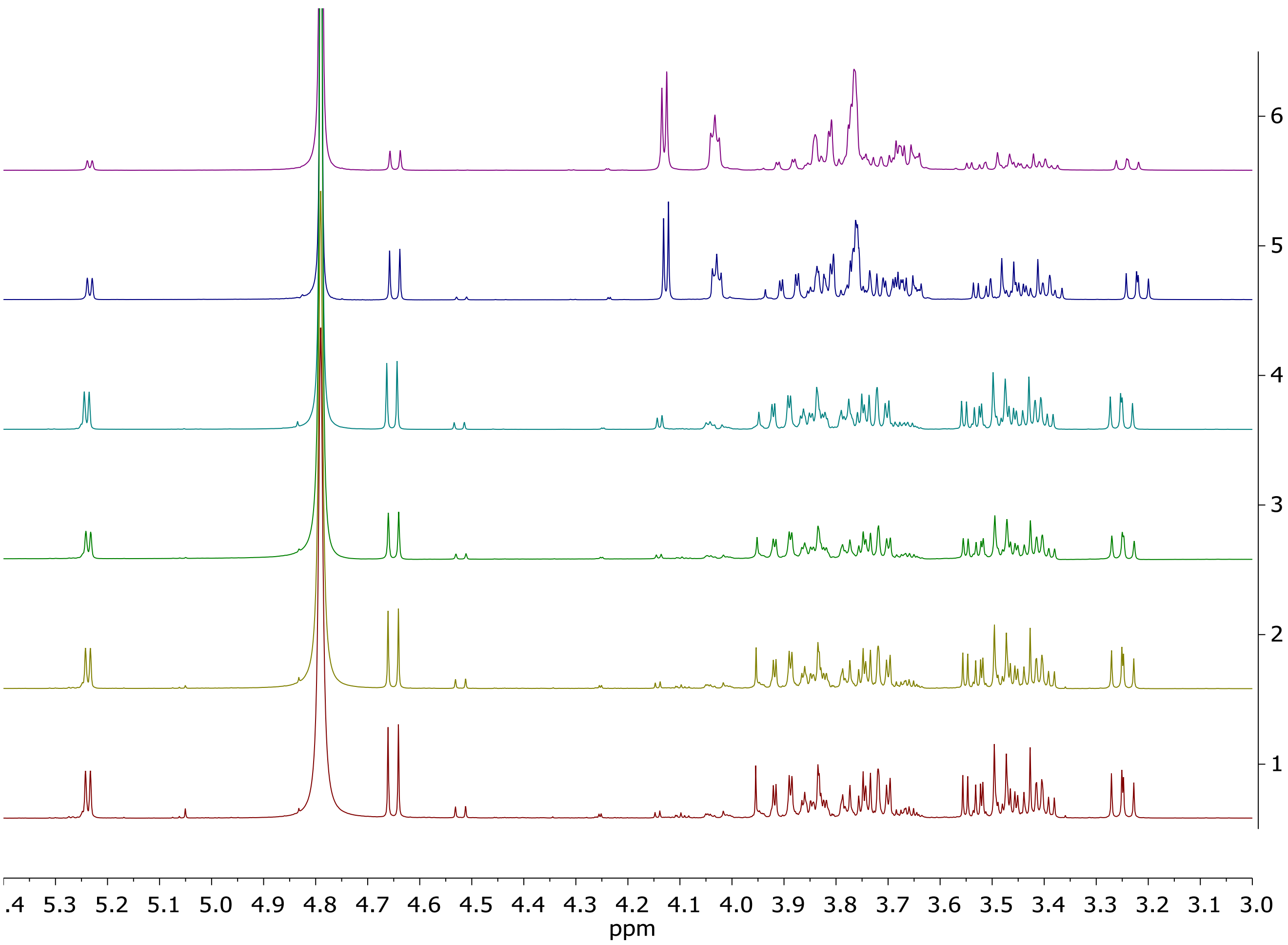


Figure S9: $^{1}H$ NMR spectra (in $D_2O$) of the crude obtained under darkness conditions at several times (red-1: 5 min, yellow-2:10 min, green-3: 15 min, cyan-4: 30 min, blue-5: 60 min, purple-6: 90 min). *Cond.:* $m_{glucose} = 250$ *mg*, $m_{cat} = 25$ *mg*, $m_{NaOH} = 55$ *mg*, $V_{H2O2} = 200$ *μL*, $V_{H2O} = 6$ *mL*.

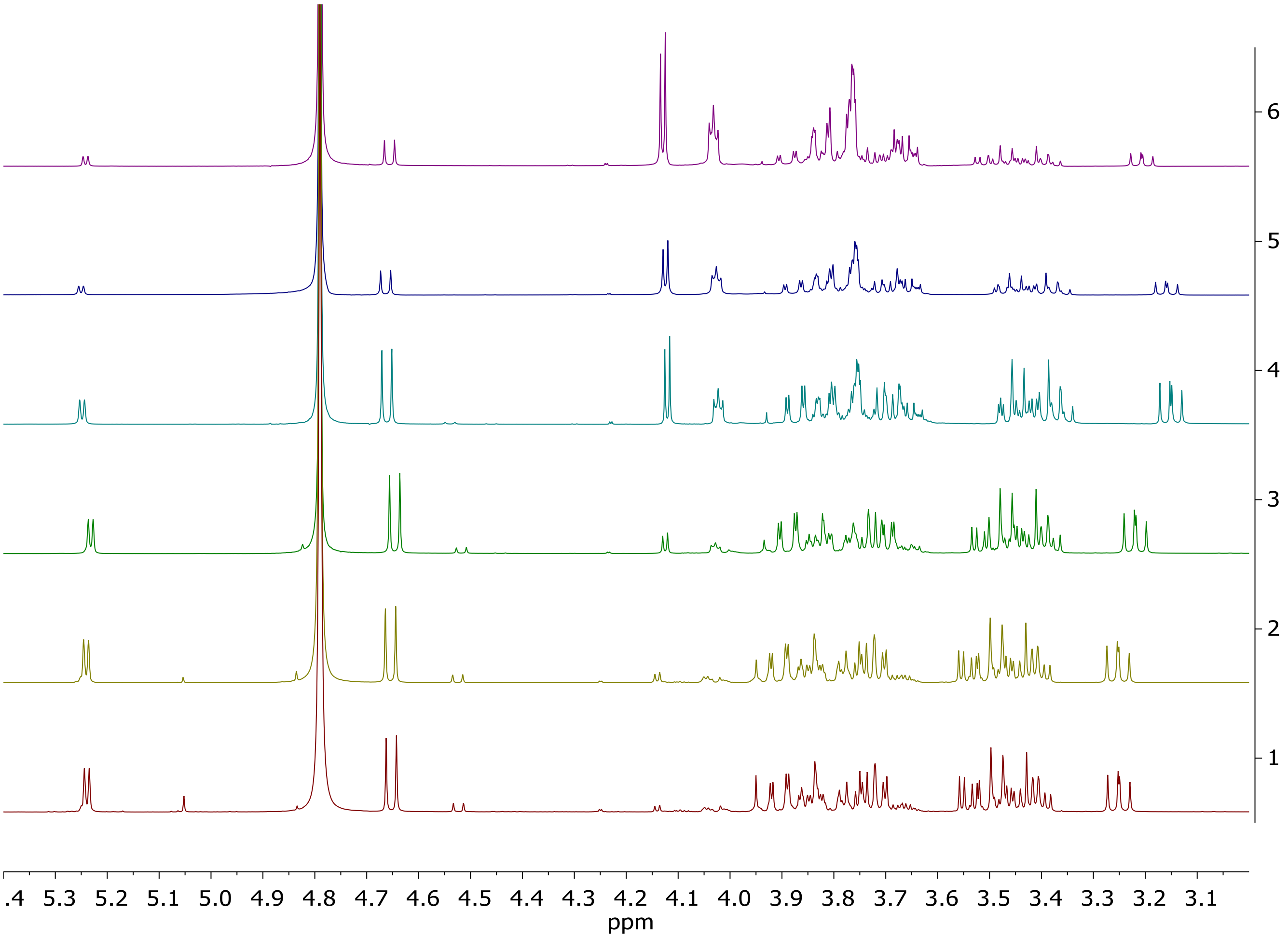


<u>Figure S10</u>: $^{1}H$ NMR spectra (in $D_2O$) of the crude obtained under heating at 35°C at several times (red-1: 5 min, yellow-2:10 min, green-3: 15 min, cyan-4: 30 min, blue-5: 60 min, purple-6: 90 min). *Cond.:* $m_{glucose} = 250$ mg, $m_{cat} = 25$ mg, $m_{NaOH} = 55$ mg, $V_{H2O2} = 200$ µL, $V_{H2O} = 6$ mL.

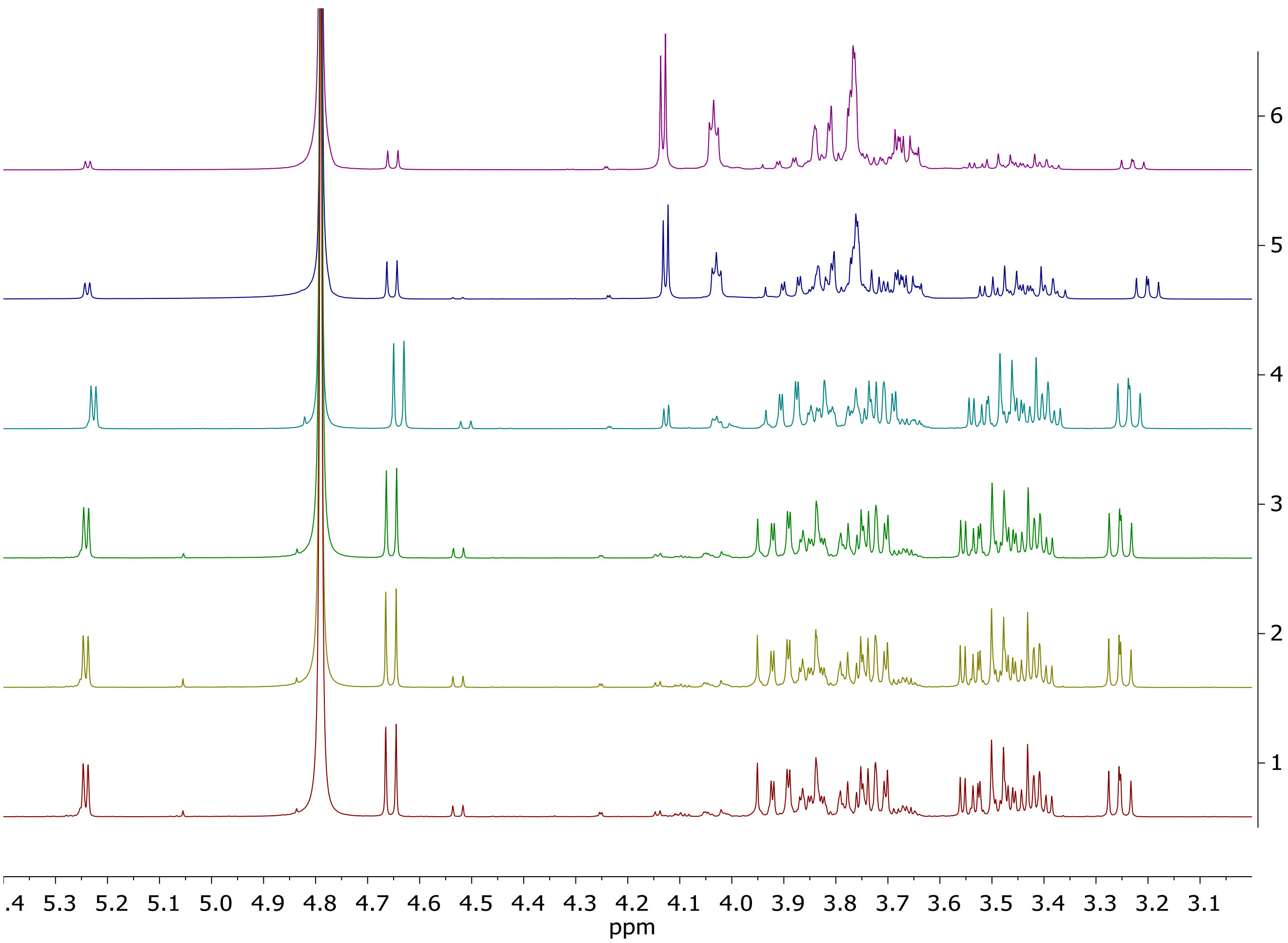


<u>Figure S11</u>: $^{1}H$ NMR spectra (in $D_2O$) of the crude obtained under A.M. 1.5 G illumination using LP475 nm filter at several times (red-1: 5 min, yellow-2:10 min, green-3: 15 min, cyan-4: 30 min, blue-5: 60 min, purple-6: 90 min). *Cond.:* $m_{glucose}$ = 250 mg, $m_{cat}$ = 25 mg, $m_{NaOH}$ = 55 mg, $V_{H2O2}$ = 200 µL, $V_{H2O}$ = 6 mL.

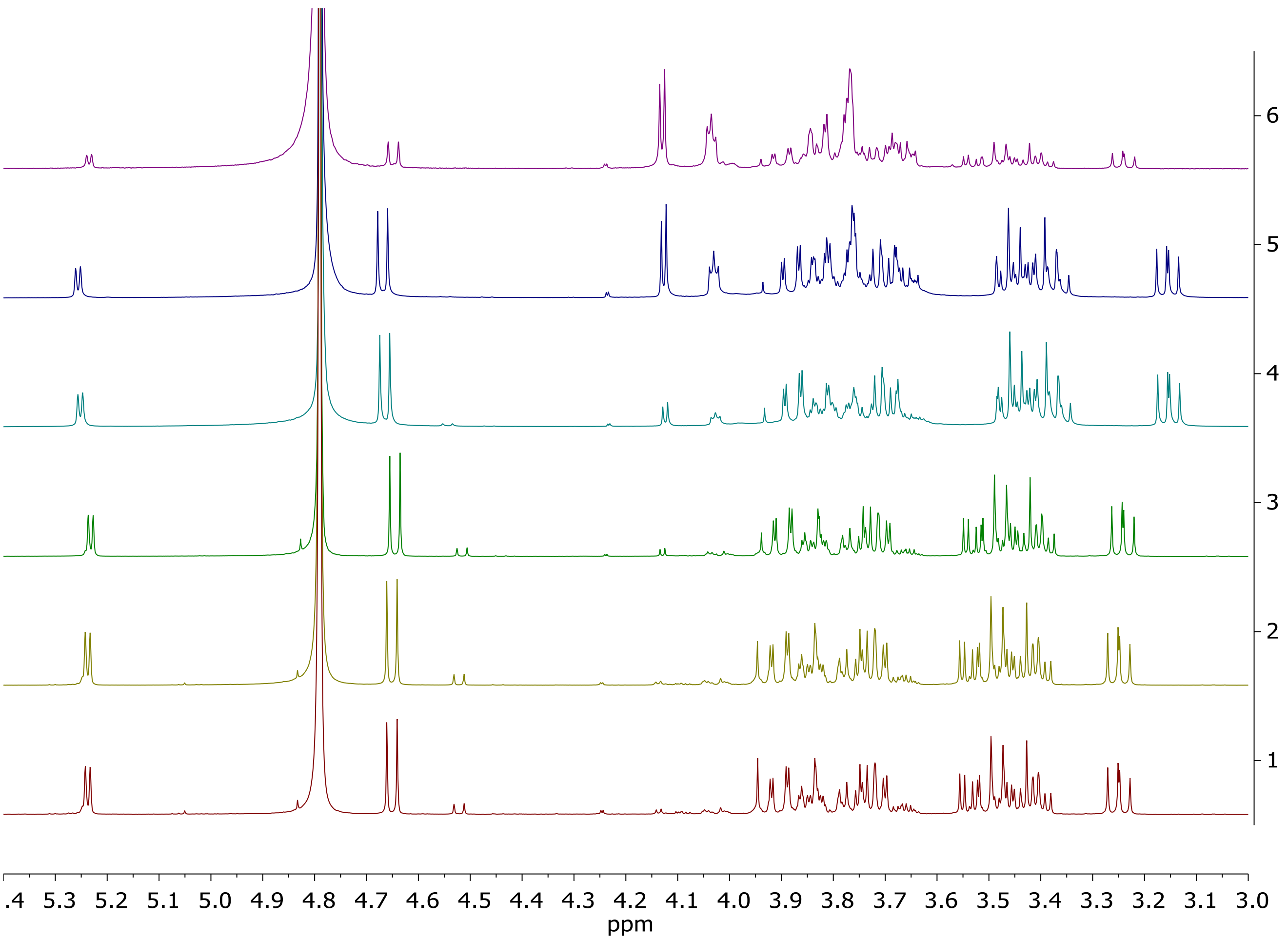


<u>Figure S12</u>: $^{1}$H NMR spectra (in $D_2O$) of the crude obtained under A.M. 1.5 G illumination using LP780 nm filter at several times (red-1: 5 min, yellow-2:10 min, green-3: 15 min, cyan-4: 30 min, blue-5: 60 min, purple-6: 90 min). *Cond.:* $m_{glucose} = 250$ *mg*, $m_{cat} = 25$ *mg*, $m_{NaOH} = 55$ *mg*, $V_{H2O2} = 200$ *µL*, $V_{H2O} = 6$ *mL*.

# 5. References


1. Rojas, R.; Ulibarri, M.Á.; Barriga, C.; Rives, V. Chromate intercalation in Ni–Zn layered hydroxide salts. *Appl. Clay Sci.* **2010**, *49*, 176–181.
2. Keller, J.; Dreisbach, F.; Rave, H.; Staudt, R.; Tomalla, M. Measurement of gas mixture adsorption equilibria of natural gas compounds on microporous sorbents. *Adsorption* **1999**, *5*, 199–214.
3. Sing, K.S. Physisorption of nitrogen by porous materials. *J. Porous Mater.* **1995**, *2*, 5–8.
4. Aramendía, M.A.; Borau, V.; Jiménez, C.; Marinas, J.M.; Ruiz, J.R.; Urbano, F.J. Comparative study of Mg/M (III)(M= Al, Ga, In) layered double hydroxides obtained by coprecipitation and the sol–gel method. *J. Solid State Chem.* **2002**, *168*, 156–161.
5. Hernandez-Moreno, M.J.; Ulibarri, M.A.; Rendon, J.; Serna, C.J. IR characteristics of hydrotalcite-like compounds. *Phys. Chem. Miner.* **1985**, *12*, 34–38.
6. Soykal, I.I.; Sohn, H.; Ozkan, U.S. Effect of support particle size in steam reforming of ethanol over Co/$CeO_2$ catalysts. *ACS Catal.* **2012**, *2*, 2335–2348.
7. Zhang, Z.-Q.; Liao, M.-C.; Zeng, H.-Y.; Xu, S.; Liu, X.-J.; Du, J.-Z.; Zhu, P.-H.; Huang, Q.-J. Temperature effect on chromium (VI) removal by Mg/Al mixed metal oxides as adsorbents. *Appl. Clay Sci.* **2014**, *102*, 246–253.
8. Rybka, K.; Matusik, J.; Kuligiewicz, A.; Leiviskä, T.; Cempura, G. Surface chemistry and structure evaluation of Mg/Al and Mg/Fe LDH derived from magnesite and dolomite in comparison to LDH obtained from chemicals. *Appl. Surf. Sci.* **2021**, *538*, 147923.
9. Gonçalves, R.G.L.; Lopes, P.A.; Resende, J.A.; Pinto, F.G.; Tronto, J.; Guerreiro, M.C.; de Oliveira, L.C.A.; de Castro Nunes, W.; Neto, J.L. Performance of magnetite/layered double hydroxide composite for dye removal via adsorption, Fenton and photo-Fenton processes. *Appl. Clay Sci.* **2019**, *179*, 105152.
10. Bouteraa, S.; Saiah, F.B.D.; Hamouda, S.; Bettahar, N. Zn-M-$CO_3$ layered double hydroxides (M = Fe, Cr, or Al): Synthesis, characterization, and removal of aqueous indigo carmine. *Bull. Chem. React. Eng. Catal.* **2020**, *15*, 43–54.
11. Amamra, S.; Djellouli, B.; Elkolli, H.; Benguerba, Y.; Erto, A.; Balsamo, M.; Ernst, B.; Benachour, D. Synthesis and characterization of Layered Double Hydroxides aimed at encapsulation of sodium diclofenac: Theoretical and experimental study. *J. Mol. Liq.* **2021**, *338*, 116677.
12. Zhang, L.-Y.; Han, Y.-L.; Liu, M.; Deng, S.-L. Ni–Al layered double hydroxide-coupled layered mesoporous titanium dioxide (Ni–Al LDH/LM-$TiO_2$) composites with integrated adsorption-photocatalysis performance. *RSC Adv.* **2023**,13, 16797–16814.
13. Myers, R.H.; Montgomery, D.C.; Anderson-Cook, C.M. *Response Surface Methodology: Process and Product Optimization Using Designed Experiments*; John Wiley & Sons: Hoboken, NJ, USA, 2016.
